# Dynamic white matter changes in recovered COVID-19 patients: a two-year follow-up study

**DOI:** 10.7150/thno.79902

**Published:** 2023-01-01

**Authors:** Sihong Huang, Xianglin Zhou, Wei Zhao, Yanyao Du, Danhui Yang, Yijie Huang, Yanjing Chen, Huiting Zhang, Guang Yang, Jun Liu, Hong Luo

**Affiliations:** 1 Department of Radiology, The Second Xiangya Hospital, Central South University, Changsha, Hunan 410011, China; 2 Department of Respiratory Medicine, The Second Xiangya Hospital, Central South University, Changsha, Hunan 410005, China; 3 MR Scientific Marketing, Siemens Healthcare Ltd., Wuhan, China; 4 Shanghai Key Laboratory of Magnetic Resonance, East China Normal University, Shanghai, China; 5 Clinical Research Center for Medical Imaging in Hunan Province, Changsha, Hunan 410011, China; 6 Department of Radiology Quality Control Center, Hunan Province, Changsha, Hunan 410011, China; 7 Department of Pulmonary and Critical Care Medicine, the Second Xiangya Hospital, Central South University, Changsha, Hunan 410011, China; 8 Hunan Diagnosis and Treatment Center of Respiratory Disease, Changsha, Hunan 410011, China

**Keywords:** recovered COVID-19 patients, white matter changes, cognitive function, two-year follow-up, inflammation factors

## Abstract

**Background and purpose:** Long COVID with regard to the neurological system deserves more attention, as a surge of treated patients are being discharged from the hospital. As the dynamic changes in white matter after two years remain unknown, this characteristic was the focus of this study.

**Methods:** We investigated 17 recovered COVID-19 patients at two years after discharge. Diffusion tensor imaging, neurite orientation dispersion and density imaging were performed to identify white matter integrity and changes from one to two years after discharge. Data for 13 revisited healthy controls were collected as a reference. Subscales of the Wechsler Intelligence scale were used to assess cognitive function. Repeated-measures ANOVA was used to detect longitudinal changes in 17 recovered COVID-19 patients and 13 healthy controls after one-year follow-up. Correlations between diffusion metrics, cognitive function, and other clinical characteristics (i.e., inflammatory factors) were also analyzed.

**Results:** Longitudinal analysis showed the recovery trends of large-scale brain regions, with small-scale brain region deterioration from one year to two years after SARS-CoV-2 infection. However, persistent white matter abnormalities were noted at two years after discharge. Longitudinal changes of cognitive function showed no group difference. But cross-sectional cognitive difference between recovered COVID-19 patients and revisited HCs was detected. Inflammation levels in the acute stage correlated positively with white matter abnormalities and negatively with cognitive function. Moreover, the more abnormal the white matter was at two years, the greater was the cognitive deficit present.

**Conclusion:** Recovered COVID-19 patients showed longitudinal recovery trends of white matter. But also had persistent white matter abnormalities at two years after discharge. Inflammation levels in the acute stage may be considered predictors of cognition and white matter integrity, and the white matter microstructure acts as a biomarker of cognitive function in recovered COVID-19 patients. These findings provide an objective basis for early clinical intervention.

## Introduction

Long COVID, also referred to as persistent post-COVID-19 syndrome, is referred to as the post COVID-19 condition by the WHO 1. Overall, more attention to this entity is needed due to the surge of treated patients being discharged from the hospital, especially those discharged from the intensive care unit 2, 3. Long COVID-19 involves persistent physical, psychological, neurological and cognitive sequelae following recovery from COVID-19 4. Core outcome sets, such as fatigue, work or occupational and study changes, have been published in *Lancet Respiratory Medicine* 5. The central nervous system (CNS) is a target of severe acute respiratory syndrome coronavirus 2 (SARS-CoV-2) infection. The impact of COVID-19 on the CNS can last for two years or even longer 6. Therefore, identifying the most suitable measure to show impacts on the CNS and understanding the potential mechanisms of long COVID may guide efforts to better manage the symptoms, improve quality of life and mitigate any economic burden.

Many post-COVID-19 symptoms, such as depression and cognitive impairment, are related to CNS injury, even in patients with no specific neurological manifestation in the acute stage, including nonhospitalized patients 7. Direct and/or indirect impacts, such as systemic inflammation, cause abnormalities after SARS-CoV-2 infection, and these effects may last for a long time 8, 9. These neurological changes have been proven by noninvasive imaging methods, such as rest-state functional MRI 10 and T1 structural imaging 11. Several previous studies with cross-sectional and longitudinal follow-up have shown changes in white matter (WM) microarchitecture in patients after COVID-19 recovery, and these changes may explain some of the post-COVID-19 symptoms 12-16. However, whether this deleterious impact can be partially reversed after two years or whether these effects persist in the long term remains to be investigated. Diffusion tensor imaging (DTI) is the most common diffusion model used to evaluate WM integrity. Neurite orientation dispersion and density imaging (NODDI), a novel diffusion model, has superiority in revealing WM changes in recovered COVID-19 patients. Therefore, we used these two diffusion models to follow up patients at one to two years after COVID-19 recovery to explore longitudinal neurobiological markers of COVID-19 injury.

In this study, we sought to address the following issues. First, we determined whether cognitive deficits or cognitive decline exist at 2 years after discharge when compared with healthy controls (HCs) and subjects followed up for one year. Second, we examined whether WM abnormalities persist after two years of follow-up and whether dynamic changes persist at one- to two-years of follow-up. Third, we sought to determine whether WM changes are associated with cognitive changes as well as other clinical characteristics, such as inflammatory factors.

## Materials and methods

This study was approved by the ethics committee of the Second Xiangya Hospital of Central South University. All participants provided written informed consent in accordance with the Declaration of Helsinki.

### Participants

**Figure 1:**
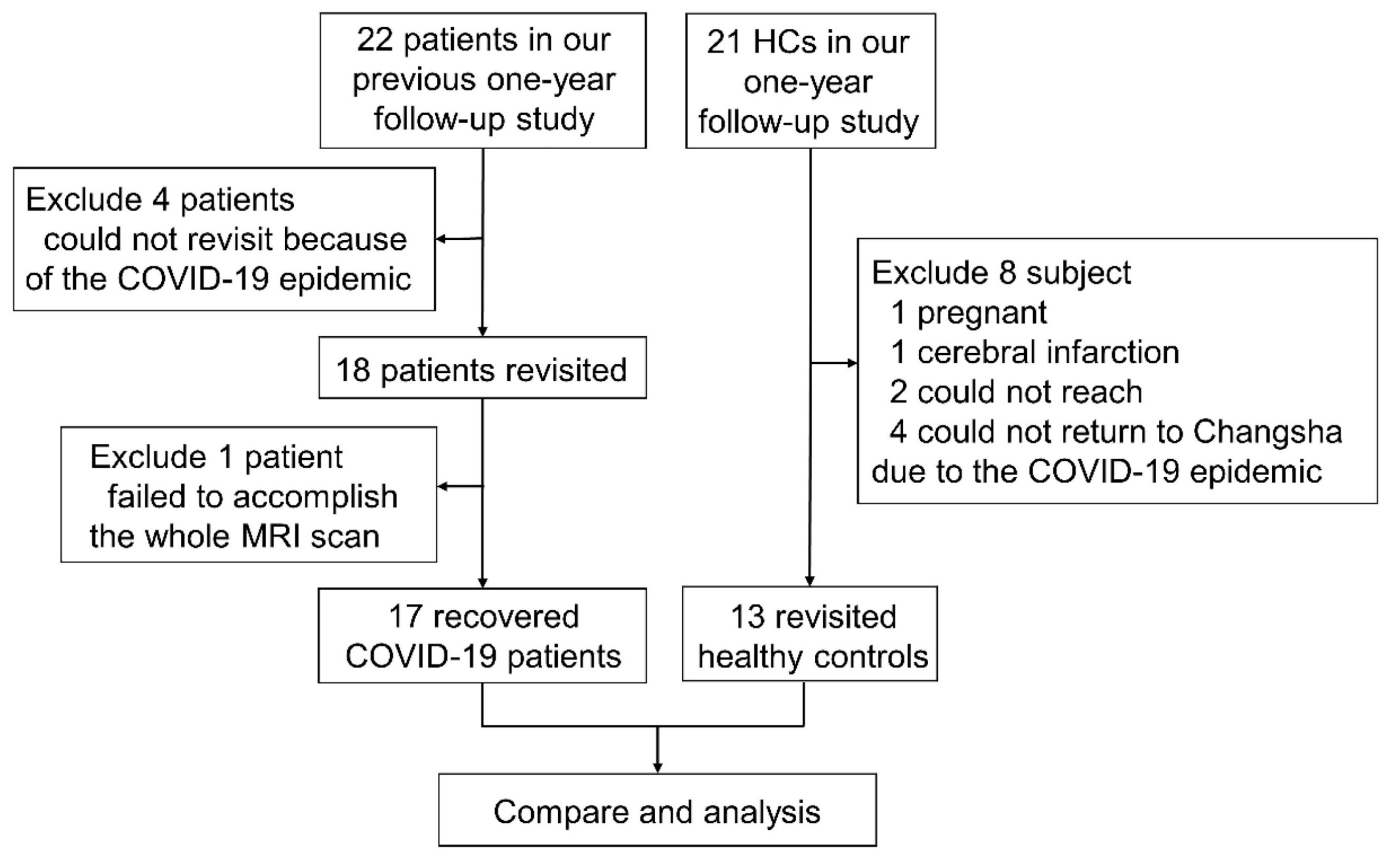
Flowchart of the study.

Twenty-two recovered COVID-19 patients who were enrolled in our previous one-year follow-up study 16 were prospectively recruited again at 2 years after discharge. In total, 18 patients were included in our study. All patients were recruited from the First Hospital of Changsha. The inclusion criteria for the recovered COVID-19 group were as follows: (1) diagnosis of COVID-19 according to guidelines of the National Health Commission 17 and discharge date between February and April 2020; (2) age greater than 18 years; and (3) willingness and ability to undergo brain MRI scanning. The exclusion criterion was a structural abnormality on traditional neuroimaging, except for WM hyperintensity. To acquire longitudinal data for HCs, twenty-one HCs who were enrolled in our previous one-year follow-up study 16 were recruited again at the same time when we examined the recovered COVID-19 patients. At that time, one HC was pregnant, one HC had recent cerebral infarction, four HCs could not return to Changsha due to the COVID-19 epidemic, and two HCs could not be reached by telephone. Therefore, 13 HCs were included. The exclusion criteria for the recovered COVID-19 patients and HCs were as follows: severe psychiatric disease (e.g., schizophrenia or depression), severe somatic disease (e.g., diabetes, uncontrolled hypertension, or heart disease), drug abuse, history of traumatic brain injury or surgery, or brain structural abnormality (e.g., encephalomalacia foci, brain infections or neoplasms) on neuroimaging, except for mild-moderate WM hyperintensity. Among the 18 discharged patients, one patient failed to undergo the whole MRI scan. Finally, 17 recovered COVID-19 patients and 13 HCs were included from Feb 2022 to May 2022. A flowchart of the subject inclusion is shown in **Figure 1**.

All subjects underwent psychiatric evaluations via face-to-face interviews conducted by trained medical staff. Information on the following clinical characteristics was collected: age; sex; education; history of sojourn; clinical type (National Health Commission guidelines: mild, moderate or severe); hospitalization days; and the presence of fever, cough, or gastrointestinal symptoms. Four inflammatory markers were collected: erythrocyte sedimentation rate (ESR); C-reactive protein (CRP); neutrophil/lymphocyte ratio (NLR) and systemic immune-inflammation index (SII) (SII = platelets * neutrophils/lymphocytes) 18. Baseline clinical characteristics and inflammatory markers were used for further analysis. The demographic characteristics and neuropsychological tests of the recovered COVID-19 patients and HCs are presented in **Table 1**.

### MRI acquisition

All MRI data were acquired using a 3-T MRI scanner (MAGNETOM Skyra, Siemens Healthcare, Erlangen, Germany) with a 32-channel head coil. All subjects were placed in the supine position with a headset or foam padding between the head and the head coil to minimize head motion. The MRI scanning sequences included T1-weighted imaging (T1WI), T2-weighted imaging (T2WI), fluid-attenuated inversion recovery (FLAIR) imaging, three-dimensional magnetization-prepared rapid acquisition gradient echo (3D MPRAGE) imaging, susceptibility weighted imaging (SWI) and diffusion MRI. All scanning parameters were the same as those in our previous study 16. T1WI, T2WI, FLAIR, MPRAGE and SWI were independently reviewed by two neuroradiologists with more than ten years of experience in neuroimaging to check for structural abnormalities. Any disagreement between the two observers was resolved by consensus.

### Neuropsychological test acquisition

All participants completed the same cognitive tests as in our previous study 16. These cognitive studies are as follows: (1) the logical memory (LM) test to test verbal episodic memory, including LM-A and LM-B; (2) digit symbol substitution test (DSST), which has been frequently used to assess participants' processing speed, sustained attention and working memory; (3) the knowledge subscale of the Wechsler Intelligence scale, which primarily measures the participant's breadth of knowledge, ability to learn and accept, and ability to understand daily things; (4) the digit span (DS) task, which was used to test verbal attention and working memory, including forward digit span (FDS) and backward digit span (BDS); (5) the word fluency test (WFT). The subjects completed these neuropsychological tests on the same day as the MRI scan. The same neuropsychologist completed all of the participants' neuropsychological scale assessments.

### Image analysis

Image processing included initial preprocessing and diffusion metric computations. Prior to preprocessing, each subject's diffusion images were visually inspected to verify them to be free from major artifacts (e.g., head motion). Motion, eddy current artifacts, and geometric distortions were corrected using the *eddy* command provided in FMRIB Software Library (FSL) 19. Using an in-house MATLAB script, the transformation matrices, output from the *eddy* command, were used to rotate the corresponding diffusion-weighting directions to match the rotation of the brain image during the motion correction process. The b0 images were extracted, and nonbrain voxels were masked out by applying the FSL *bet* command to the subject's b0 image. Then, four DTI metrics (fractional anisotropy (FA), radial diffusivity (RD), axial diffusivity (AD) and mean diffusivity (MD)) were calculated by the FSL *dtifit* command. Three NODDI parameters (orientation dispersion index (ODI), volume fraction of intracellular water (V_ic_) and volume fraction of the isotropic diffusion compartment (V_iso_)) were calculated using the open-source tool AMICO (https://github.com/daducci/AMICO) 20.

### Tract-based spatial statistics (TBSS) analysis

TBSS, a whole-brain analysis that combines the strengths of voxel-based analyses and tractography-based analyses, was performed using the FSL toolbox TBSS. A common whole-brain white-matter skeleton was extracted in the standard Montreal Neurological Institute (MNI) space to minimize the partial volume effects at a finite imaging resolution. The WM skeleton included only voxels in the center of WM tracts and excluded edge voxels, which may be contaminated with signals from the nearby anatomy. Cross-sectional comparison involved a matrix design of an unpaired two-sample t test with sex as a covariate and longitudinal comparison a matrix design of a paired two-sample t test using FSL. Within the WM skeleton, nonparametric permutation-based statistics were performed using the FSL *randomize* command for voxelwise statistical analyses, and sex was used as a covariant. Threshold-free cluster enhancement 21 and 5000 permutations were utilized to obtain a corrected *p* value. WM voxels were considered significant at a corrected *p* value < 0.05 after being adjusted for multiple comparisons by controlling the familywise error (FWE) rate.

### Post hoc region-of-interest (ROI) analysis

To produce aggregate results at the subject level, post hoc ROI analyses were performed. For each subject, the mean of each diffusion metric was computed in regions that tested significant with TBSS. Unpaired two-sample t tests and Kruskal‒Wallis tests (nonnormally distributed samples) were carried out for the cross-sectional comparison of diffusion metrics. Paired two-sample t tests and Wilcoxon tests (nonnormally distributed samples) were used for longitudinal comparison of diffusion metrics. Boxplots were used with subject means plotted based on group membership. The anatomical interpretation of the ROI was based on the “JHU ICBM-DTI-81 White-Matter Labels” provided in FSL after skeletonization.

### Statistical analysis

Demographic and clinical characteristics and aspects of the neuropsychological data were analyzed using IBM SPSS Statistics 24.0. A chi-square test was performed for sex. Unpaired two-sample t tests and Kruskal‒Wallis tests (nonnormally distributed samples) were carried out for age and education. In addition, unpaired two-sample t tests and Kruskal‒Wallis tests (nonnormally distributed samples) were conducted for cross-sectional comparison of neuropsychological tests. Repeated-measures ANOVA was used to detect longitudinal comparison of neuropsychological tests. Partial correlations were conducted between diffusion parameters and neuropsychological tests using sex as a covariate between two-year follow-up patients and HCs. Bivariate correlation analysis (Pearson correlation for normally distributed samples and Spearman correlation for nonnormally distributed samples) of diffusion parameters, neuropsychological test scores and clinical characteristics was conducted among recovered COVID-19 patients. Correlations were corrected for multiple comparisons using an FWE correction.

### Data availability

The data that support the findings of this study are available from the corresponding author upon reasonable request.

## Results

### Demographic and clinical characteristics

The study included 17 (male: 9; female: 8) recovered COVID-19 patients and 13 (male: 1; female: 12) HCs. Our study included four datasets: the HC group in 2021 (HC1, N = 13); the HC group in 2022 (HC2, N = 13); recovered patients at one year after discharge (PT1, N = 17) and recovered patients at two years after discharge (PT2, N = 17). Comparison of the characteristics between PT2 and HCs is presented in **Table 1**. There were no statistically significant differences between the patients and HCs with regard to age, or education, but sex ratio had group difference (*p* = 0.017). Two patients had complications: one had sepsis and multiple organ dysfunction syndrome (MODS), and the other had acute respiratory distress syndrome (ARDS). Comparison between PT1 and PT2 is presented in **Table 1**. One patient needed ventilatory assistance in the acute stage. There were no ESR data in 8 patients and we did two treatments in the correlation analysis: excluding missing data and filling them with the mean value. In total, 11/17 (64.71%) patients had neurological symptoms at one year after discharge, with 13/17 (76.47%) having neurological symptoms at two years after discharge.

### Diffusion metrics

**Figure 2:**
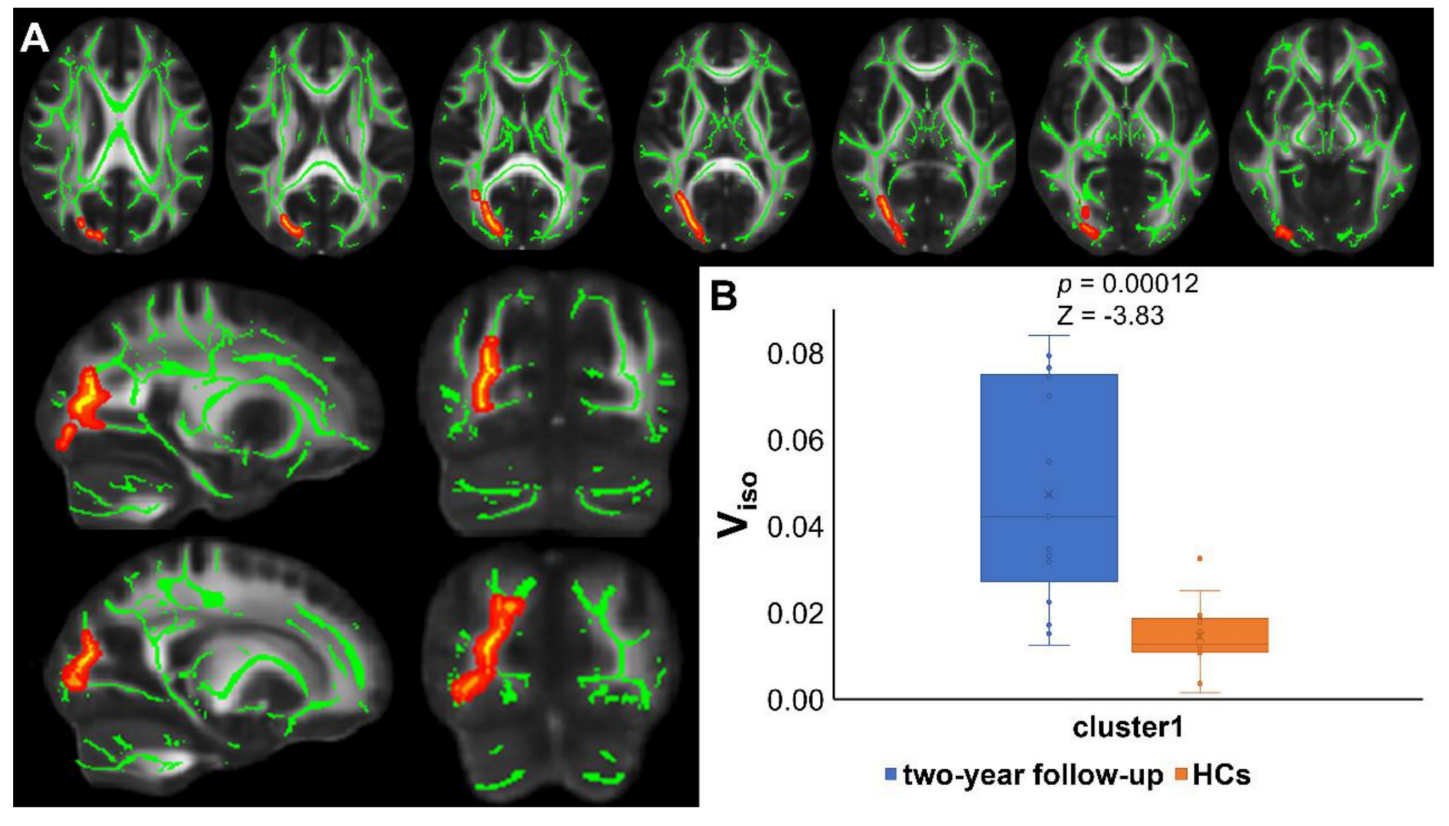
** Results of TBSS analysis and post hoc ROI analysis (PT2 vs HC2).** (A) Tract-based spatial statistics (TBSS) results for V_iso_ between recovered COVID-19 patients and HCs. The TBSS analyses revealed increased V_iso_ in patients than in controls. Green represents white matter skeleton. Red-yellow represents areas of significant differences. See **Table 2** for detail information of these tracts. (B) Post hoc region-of-interest (ROI) analysis results. Clusters are significant tracts in TBSS. The blue boxes represent recovered COVID-19 group, and the orange boxes represent HCs. PT2, recovered patients at two years after discharge; HC2, healthy controls in 2022; V_iso_, volume fraction of isotropic diffusion compartment.

**Figure 3:**
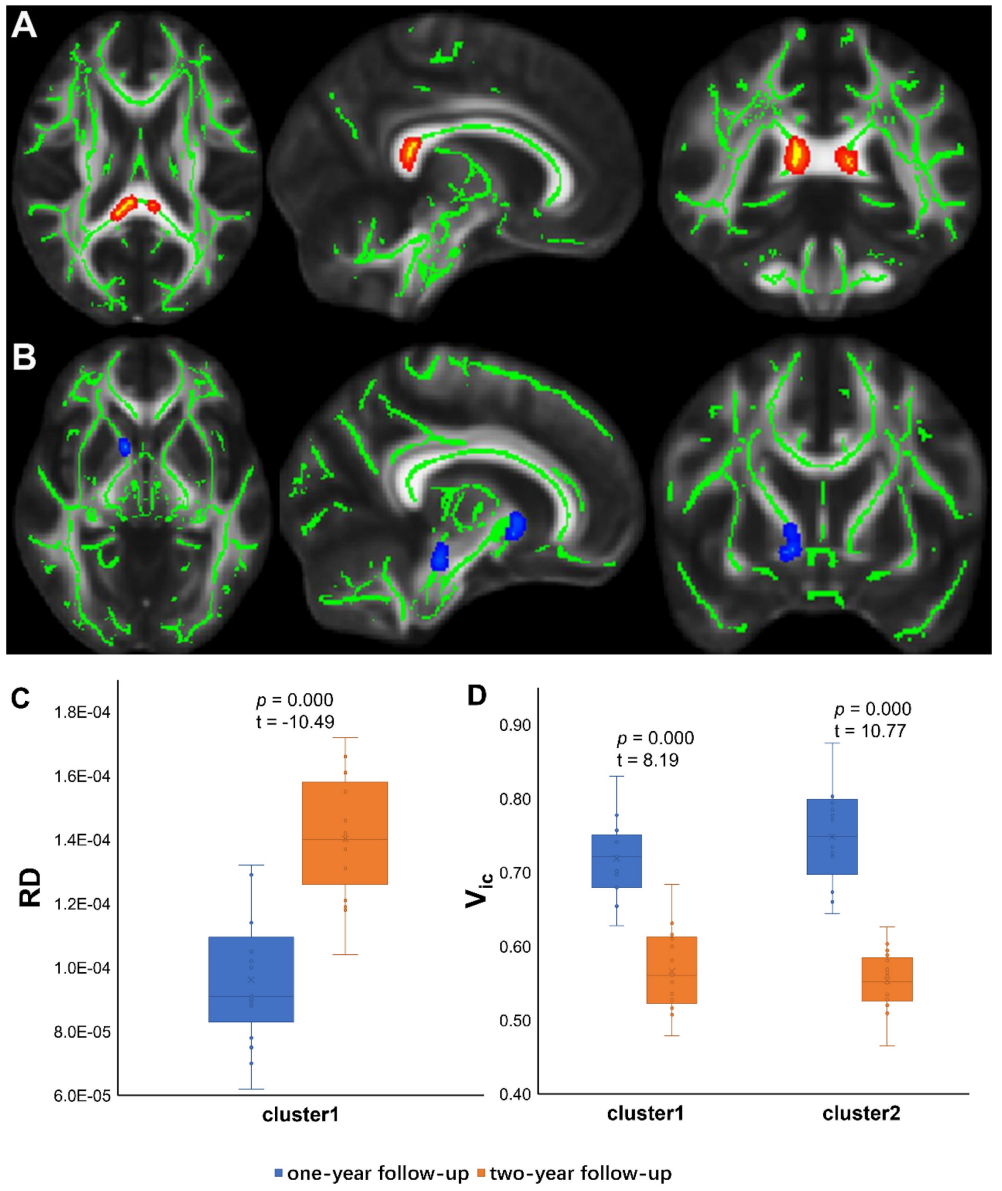
** TBSS Results of deteriorate diffusion metrics and post hoc ROI analysis (PT1 vs PT2).** (A) Tract-based spatial statistics (TBSS) results for RD between PT1 and PT2. The TBSS analyses revealed increased RD in patients two years after discharge than in patients one year after discharge. Green represents white matter skeleton. Red-yellow represents areas of significant differences. See **Table 2** for detail information of these tracts. (B) TBSS results for V_ic_ between PT1 and PT2. The TBSS analyses revealed decreased V_ic_ in patients two years after discharge than in patients one year after discharge. Green represents white matter skeleton. Blue-light blue represents areas of significant differences. See **Table 2** for detail information of these tracts. (C) (D) Post hoc region-of-interest (ROI) analysis results. Clusters are significant tracts in TBSS. The blue boxes represent PT1, and the orange boxes represent PT2. PT1, recovered patients at one years after discharge; PT2, recovered patients at two years after discharge; RD, radial diffusivity; Vic, volume fraction of intracellular water.

**Figure 4:**
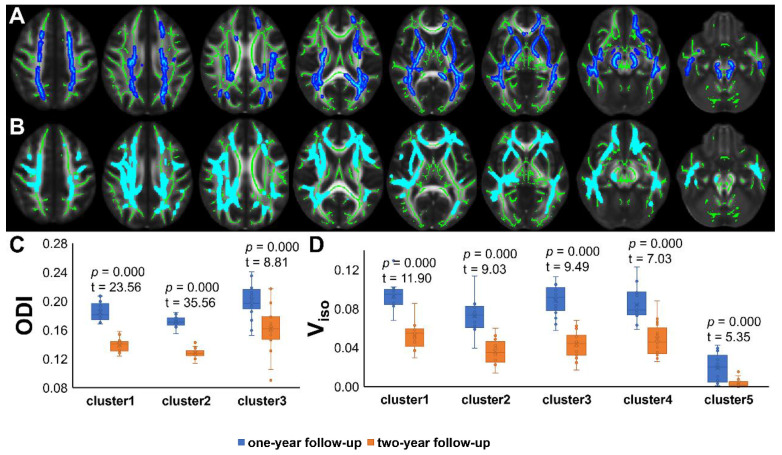
** TBSS Results of recovered diffusion metrics and post hoc ROI analysis (PT1 vs PT2).** (A)/(B) Tract-based spatial statistics (TBSS) results for ODI/V_iso_ between PT1 and PT2. The TBSS analyses revealed decreased ODI/V_iso_ in patients two years after discharge than in patients one year after discharge. Green represents white matter skeleton. Blue-light blue represents areas of significant differences. See **Table 2** for detail information of these tracts. (C) (D) Post hoc region-of-interest (ROI) analysis results. Clusters are significant tracts in TBSS. The blue boxes represent PT1, and the orange boxes represent PT2. PT1, recovered patients at one years after discharge; PT2, recovered patients at two years after discharge; ODI, orientation dispersion index; V_iso_, volume fraction of isotropic diffusion compartment.

TBSS analyses revealed higher V_iso_ in PT2 than in HC2, showing an increase in the free water volume fraction. Abnormal diffusion metrics were detected in the posterior thalamic radiation R (PTR) (**Figure 2**). Higher RD and lower ODI, V_ic_ and V_iso_ values were detected in PT2 than in PT1. Abnormal diffusion metrics were detected in the following regions: the corpus callosum (CC), bilateral corona radiata (CR), cerebral peduncle (CP), internal capsule L (IC), PTR, sagittal stratum (SS), external capsule L (EC), superior longitudinal fasciculus (SLF), and corticospinal tract L (CST) (**Figure 3 and Figure 4**). Further details of the significant results are shown in **Table 2**. And boxplots in **Figures 2-4** showed the results of post hoc ROI analysis with detail *p* and Z/t values.

### Neuropsychological test results and correlation analysis

**Figure 5:**
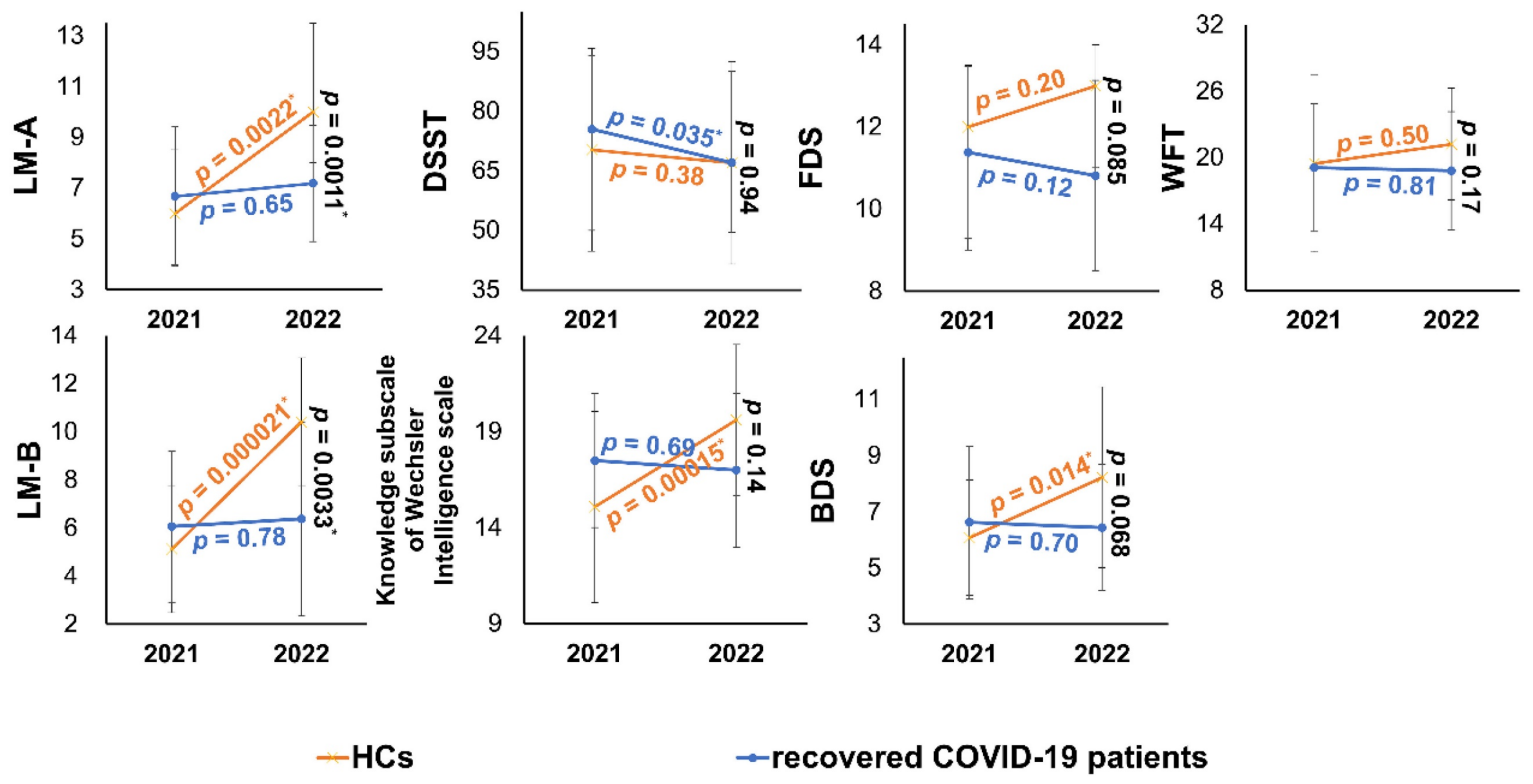
** Cognitive function changes over 1 year in recovered COVID-19 patients and HCs.** In the one- to two-year follow-up, the cognition function of recovered COVID-19 patients showed flat or declining trends (in blue). From 2021 to 2022, the cognitive function of HCs showed an improved trend in 13 HCs except for DSST (in orange). The endpoints of the line segments are mean or median, and the error bars are standard deviations or quartiles. ^*^ *p* < 0.05. More details of cross-sectional comparisons were shown in **Table 1**. HCs, healthy controls; LM, logical memory task; DSST, digital symbol substitution test; DS, digit span task; FDS, forward digit span; BDS, backward digit span; WFT, Word fluency test.

All neuropsychological test datasets for one recovered COVID-19 patient were lost at the one-year follow-up. Longitudinal changes of cognitive function showed no group difference using repeated-measures ANOVA (**Table 1**). However, LM-A, and LM-B scores were significantly different between PT2 and revisited healthy controls (HC2) (**Table 1**). The cognitive changes over 1 year are presented in **Figure 5**.

**Figure 6:**
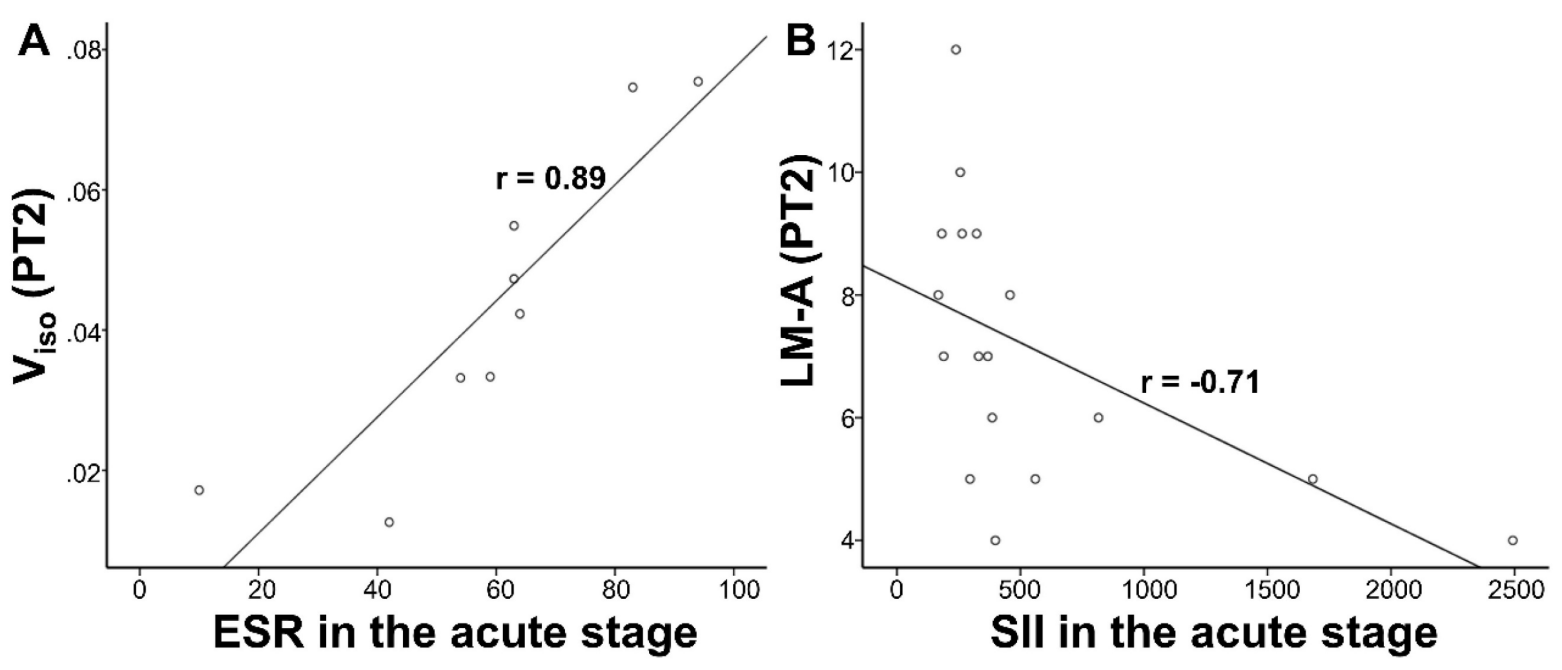
** Correlations results.** (A) V_iso_ were positively correlated with ESR in the acute stage within the PT2. (B) LM-A scores were negatively correlated with SII in the acute stage within the PT2. V_iso_, volume fraction of isotropic diffusion compartment; ESR, erythrocyte sedimentation rate; SII, systemic immune-inflammation index; LM, logical memory task; PT2, recovered patients at two years after discharge.

There was no significant correlation in longitudinal comparison (PT1 vs. PT2). In the cross-sectional comparison (PT2 vs. HC2), LM-A scores correlated negatively with CRP (*p*=0.024, *r*=-0.546) and SII (*p*=0.0015, *r*=-0.706) in the acute stage, V_iso_ correlated positively with ESR (exclude missing data: *p*=0.0015, *r*=0.886; fill missing data with the mean value: *p*=0.019, *r*=0.562) within the PT2 group. LM-A scores correlated negatively with V_iso_ detected by partial correlation in the whole group of PT2 and HC2 (**Table 3**). However, after multiple comparison correction, only the correlation between Viso and ESR (exclude missing data) as well as LM-A and SII remained in the recovered COVID-19 group. The significant correlations after FWE correction in **Table 3** are presented in **Figure 6**.

## Discussion

In the present study, we comprehensively investigated WM changes in recovered COVID-19 patients at two years after discharge using conventional DTI metrics and novel NODDI models. To the best of our knowledge, this is the first study to investigate such WM microstructure changes at a two-year follow-up. Our longitudinal analysis showed the recovery trends of large-scale brain regions, with small-scale brain region deterioration from one year to two years after SARS-CoV-2 infection. However, persistent WM abnormalities compared with HCs were found at two years after discharge. Cross-sectional cognitive deficits were present in recovered COVID-19 patients compared to HCs. But longitudinal changes of cognitive function showed no group difference between patients and HCs. Inflammation levels in the acute stage correlated positively with white matter abnormalities and negatively with cognitive function. Moreover, the more abnormal the WM was at two years, the greater was the cognitive deficit that may be present.

Among the 7 diffusion parameters, only V_iso_ showed statistical significance after correction for multiple comparisons in cross-sectional comparison, indicating that WM microstructural changes in recovered COVID-19 patients may be subtle. The NODDI was a better diffusion model for demonstrating these subtle changes in the WM. At two years after recovery, the patients had less voxels with WM abnormalities than in our previous work 16, which showed a gradual recovery of WM. Our findings are supported by the results of a previous study, though larger significant brain regions were detected because of the shorter follow-up period 22. Although we cannot directly use the size of the voxels to show the abnormal progression or recovery of the WM due to the change in the subjects, the results offer a clue to the recovery trend from the subacute phase to the chronic phase after discharge, consistent with our longitudinal results. As inflammatory storms in the acute stage acted as the indicator of WM abnormalities in our research, we can predict the white matter integrity of COVID-19 patients based on the severity of inflammation in the acute stage. A higher V_iso_ may be caused by a lower intraneurite and/or extraneurite water proportion. Therefore, our results indicate that decreased neurosupportive cells and/or reduced neurosensity may be affected by the effect of inflammation, and the correlation has been investigated in a previous study 23.

RD reflects perpendicular diffusion toward membranes, and it is increased when these two vectors perpendicular to the fiber direction are increased, indicating damaged myelination 24, 25. The dynamic change in RD showed long-term lack of recovery and more serious WM abnormalities in recovered COVID-19 patents in limited voxels of the splenium of the CC. V_ic_, a potential proxy for axonal density measurements, may be explained by edema and axonal beading followed by apoptosis 26. In our study, V_ic_ was significantly lower in PT2 than in PT1, with a decrease in neurite density in the anterior corona radiata and cerebral peduncle of the right hemisphere during longitudinal follow-up. Since no significant results for V_ic_ were found in the comparison of PT2 and HC2, we infer that abnormal axonal density of CR, GCC and SLF in our previous study 16 gradually returned to normal during one- to two-year follow-up, while axonal density of CP and IC decreased from higher than healthy controls to lower than healthy controls. Of course, this inference requires further research to prove. The dynamic change in the above two diffusion parameters revealed a small region of WM microstructural deterioration over time. Although there is weak evidence for direct viral invasion, the SARS-CoV-2 viral proteins detected in the brainstem made us unable to rule out the long-term effects of direct virus invasion 27. Additionally, the persistence of SARS-CoV-2 RNA in transplanted lung tissue 28 prevents ruling out the possibility of RNA in other deep tissues persistently, which requires further anatomical confirmation. The longitudinal effect of hypoxia/ischemia and systemic inflammation may be the causes of WM deterioration 9, 29. Additionally, COVID-19-related neurodegeneration can be reflected by WM abnormalities 11, 30. Whether this deterioration can be partially reversed or whether it persists in the long term remains to be investigated through additional follow-up.

Decreased ODI indicated that the WM fibers tended to be parallel and organized, and decreased V_iso_ indicated decreased free water content within the tissue. Such a decrease in free water may be caused by the increase in intraneurite and/or extraneurite water. Moreover, enlargement of neurosupportive cells such as astrocytes can lead to a decrease in free water. For the changes reported, each person was used as their control subject; thus, these findings truly strongly indicate the recovery trends between one- and two-year follow-up. Additionally, these WM tracts detected in our study, such as CC, CST, and IC, were consistent with a one-month follow-up study 22. That study indicated edema in the subacute stage, and our study revealed a dynamic reduction in the effects of chronic inflammation and blood‒brain barrier dysfunction. Previous longitudinal studies have also reported this multidirectional change in WM 15, indicating a certain heterogeneity in the prognosis of COVID-19 patients, and the underlying mechanism needs to be verified with larger sample data. The WM hyperintensity of the 17 patients in our study exhibited no significant change, as assessed by two experienced radiologists. Therefore, the advanced DWI model, especially the NODDI model, is able to provide more detailed information about the prognosis of recovered COVID-19 patients.

Though longitudinal changes of cognitive function showed no group difference. Compared with revisited HCs, recovered COVID-19 patients at two years after discharge showed lower LM-A and LM-B scores. On the one hand, our cross-sectional results may indicate subtle trend of weaker verbal episodic memory 31 detected by LM compared to revisited HCs. We also observed slight longitudinal reductions in several cognitive function test scores of recovered COVID-19 patients with no group differences. Worse cognitive function and a higher risk of dementia have been revealed in several studies, yet mostly with a relatively short follow-up period 7, 9, 11, 32, 33. Longer follow-up period should be conducted to demonstrate the evolution of cognitive function in patients who have recovered from COVID-19. Neurovascular elements, hypoxia/ischemia, and inflammation might also be involved in the development of post-infection cognitive decline 29, 30. Additionally, Steve Reiken et al. described the neuropathology of cognitive deficits after SARS-CoV-2 infection 34. The negative correlations between cognitive tests (LM-A) and inflammatory factors (CRP and SII) in our results revealed the effects of cytokine storms on long-term cognitive decline. On the other hand, better performance of HC2 than HC1 may be the main reason for the cross-sectional results of cognitive function, high occupation attainment, high level of physical and social activity, which could improve cognitive performance detected in other research 35, maybe the reasons for our results, but our sample size limited the possibilities for further quantification or doing sub-group analysis. Also, learning effects of repeated measures on neuropsychological tests played an important role. The correlation between V_iso_ and cognitive function indicate the intracranial microstructural changes for cognitive decline and provide objective neurobiological markers.

There were several limitations in the present study. First, our study had a small sample size. Moreover, missing data were assumed to be missing completely at random. Due to the limited number of confirmed patients in Changsha and the fact that many patients were not natives of Changsha, few patients were recruited for our one-year and two-year follow-up study. To improve the reliability of the results, we used multiple diffusion models and metrics to more comprehensively display WM changes using voxel-based methods. Strict statistical analysis and correction were also performed. Nevertheless, multicenter data should be collected for further research and verification. Second, we also performed longitudinal data collection of HCs for better comparison. However, only a portion of the previous HCs were able to be included, and we could not match the sex ratio between the experimental group and the control group. So, we used sex as a covariant in the TBSS and correlation analysis. And then, we cannot avoid learning effects of repeated measures on neuropsychological tests. Longitudinal collection of both recovered COVID-19 patients and HCs helped us neutralize the differences between the groups caused by the learning effects. Furthermore, all participants with COVID-19 in our study were hospitalized; thus, our findings may not represent the full spectrum and severity of COVID-19. Collection of multicenter data in our future work will provide more possibilities for our research, such as subgroup analyses. Last, we did not include control groups such as those with infection by other viruses (e.g., influenza A or influenza B), and we thus cannot state whether the diffusion parameter abnormality or cognitive deficits are specific for COVID-19. We attempted to recruit recovered influenza patients, but the outbreak and spread of COVID-19 (influenza patients avoid hospital admissions or the use of masks) led to an extremely low number of confirmed patients in the hospital. Most of the confirmed patients were children who could not be matched for age with recovered COVID-19 patients. Therefore, in the present study, only HCs were used as the control group.

In conclusion, recovered COVID-19 patients showed longitudinal recovery trends of WM, but also had persistent WM abnormalities at two years after discharge. Inflammation levels in the acute stage may be considered predictors of cognition and WM integrity, and the WM microstructure acts as a biomarker of cognitive function in recovered COVID-19 patients. These findings provide an objective basis for early clinical intervention.

## Figures and Tables

**Table 1:** Demographic and neuropsychological tests of recovered COVID-19 patients and HCs

	COVID-19		HCs		p	p
	**one-year follow up (PT1)**	**two-year follow up (PT2)**	**2021 (HC1)**	**2022 (HC2)**	**PT2-HC2**	**ANOVA**
N	17		13			
Sex	M:9; F:8		M:1; F:12		0.017^a^	
Education(y)	12 (10.5; 16)		12 (10.5; 15)		0.71^b^	
Age(y)	53.82±10.24	54.82±10.24	50.46±12.61	51.46±12.61	0.43^c^	
Clinical type						
Moderate	10/17					
Severe	7/17					
Inflammatory markers (acute stage)						
ESR (mm/h)	59.11±23.91					
CRP (mg/L)	36.13±28.07					
NLR	2.39 (1.81; 3.07)					
SII	330.45 (247.79; 509.09)					
Hospitalization days	16 (13; 30.5)					
Follow-up days	345 (329.5; 354)	793 (777.5; 797)				
Neurological symptoms						
Fatigue	2 (11.76%)	3 (17.65%)				
Headache	4 (23.53%)	5 (29.41%)				
Myalgia	4 (23.53%)	3 (17.65%)				
Smell loss	1 (5.88%)	2 (11.76%)				
Taste loss	0	0				
White matter hyperintensity (Fazekas scale)						
0/1/2	8/6/3	8/6/3				
Neuropsychological tests						
LM-A	6.88±2.75	7.12±2.23	6.38±3.23	10.54±2.93	**0.0011** ^c^	0.11^d^
LM-B	5.56±3.14	5.53±4.14	4.62±2.50	9.92±3.04	**0.0033** ^c^	0.13^d^
DSST	70.81±23.21	67.24±23.00	70.23±25.53	66.54±29.27	0.94^c^	0.99^d^
Knowledge subscale of Wechsler Intelligence scale	17.56±4.18	17.18±4.61	15.08±4.97	19.62±3.93	0.14^c^	0.89^d^
FDS	11.5 (11; 12.75)	11 (9; 13)	12 (9; 13.5)	13 (11; 14)	0.085^b^	0.42^d^
BDS	6.63±2.73	6.35±2.21	6.08±2.06	8.23±3.22	0.068^c^	0.48^d^
WFT	19.09±5.77	18.76±5.09	19.44±7.99	21.31±4.52	0.17^c^	0.57^d^

Bold font *p* < 0.05. ESR, erythrocyte sedimentation rate; CRP, C-reactive protein; NLR, neutrophil/lymphocyte ratio; SII, systemic immune-inflammation index; LM, logical memory task; DSST, digital symbol substitution test; DS, digit span task; FDS, forward digit span; BDS, backward digit span; WFT, Word fluency test.

**Table 2:** Anatomical regions of tract-based spatial statistics results

	Cluster index	Anatomical regions	Voxels	min p	X	Y	Z
**PT2 vs HC2**							
V_iso_	1	Posterior thalamic radiation R	919	0.026	62	52	82
**PT1 vs PT2**							
RD	1	Splenium of corpus callosum	173	0.003	82	88	87
ODI	1	Splenium of corpus callosum	7858	0	131	85	69
		Corticospinal tract L					
		Cerebral peduncle L					
		Internal capsule L					
		Corona radiata L					
		Posterior thalamic radiation L					
		Sagittal stratum L					
		External capsule L					
		Superior longitudinal fasciculus L					
	2	Superior cerebellar peduncle R	5834	0	49	92	64
		Cerebral peduncle R					
		Internal capsule R					
		Corona radiata R					
		Posterior thalamic radiation R					
		Sagittal stratum R					
		External capsule R					
		Superior longitudinal fasciculus R					
	3	Anterior corona radiata R	272	0.013	65	154	73
V_ic_	1	Cerebral peduncle R	57	0.047	78	99	55
	2	Anterior limb of internal capsule R	38	0.041	78	133	73
V_iso_	1	Corpus callosum	8196	0	41	100	53
		Cerebral peduncle R					
		Internal capsule R					
		Corona radiata R					
		Sagittal stratum R					
		External capsule R					
		Superior longitudinal fasciculus R					
	2	Anterior limb of internal capsule L	2281	0	107	152	55
		Anterior corona radiata L					
	3	Posterior thalamic radiation L	616	0	131	119	48
	4	Superior longitudinal fasciculus L	148	0.009	129	71	107
	5	External capsule L	169	0.011	118	138	65

PT2, recovered patients at two years after discharge; HC2, healthy controls in 2022; PT1, recovered patients at one years after discharge; V_iso_, volume fraction of isotropic diffusion compartment; RD, radial diffusivity; ODI, orientation dispersion index; V_ic_, volume fraction of intracellular water.

**Table 3:** Correlation results

	X	Y	r	p
Pearson correlation	ESR (exclude missing data)	Viso	0.886	**0.0015**
	ESR (fill missing data with the mean value)	Viso	0.562	0.019
	CRP	LM-A	-0.546	0.024
Spearman correlation	SII	LM-A	-0.706	**0.0015**
Partial correlation	Viso	LM-A	-0.387	0.038

Bold font *p* < 0.0042 (0.05/12) for FWE correction. ESR, erythrocyte sedimentation rate; V_iSO_, volume fraction of isotropic diffusion compartment; CRP, C-reactive protein; LM, logical memory task; SII, systemic immune-inflammation index; FWE, familywise error.

## Abbreviations

COVID-19: coronavirus disease 2019
DTI: diffusion tensor imaging
NODDI: neurite orientation dispersion and density imaging
SARS-CoV-2: severe acute respiratory syndrome coronavirus 2
TBSS: tract-based spatial statistics
